# Prevalence of a dual diagnosis of mental illness and substance use disorder in Ontario, Canada: a retrospective cohort study using linked administrative data

**DOI:** 10.23889/ijpds.v9i5.2838

**Published:** 2024-09-18

**Authors:** Rachel Huck

**Affiliations:** 1 Office for National Statistics

## Objective

Data linkage is increasingly used to generate research-ready datasets to help inform decision makers. Although use of linked data is increasing and accepted as an efficient way to generate new insights, linkage methods and error can have a significant impact on the subsequent analysis and interpretation of the results. Current linkage services overcome this by spending time explaining the linkage to the user. However, as the service demand increases and a generalised approach adopted, ensuring the information is available and understood is a growing challenge. Providing and presenting the information in an accessible way has therefore become a new problem for the data linkage experts.

## Approach

User researchers are working with groups of non-linkage experts using linked data to collate and understand feedback on:

what information researchers expect.
the information researchers find useful.
how researchers interpret and use the linkage information to inform their analysis.

## Results

Findings to date will be presented by exploring how information should be presented and what information researchers are able to interpret and use as part of their analysis.

## Conclusions/Impact

Findings will provide guidance as to the information which should be included with a linkage output and the structure most accessible to the research community using linked data. Therefore, the research will help shape how the data linkage service is structured in the future by ensuring researchers can access information they require to interpret linked data and therefore use it appropriately. Ultimately, the feedback will help deliver data linkage services on a large scale.

